# Repurposing existing drugs for monkeypox: applications of virtual screening methods

**DOI:** 10.1007/s13258-023-01449-8

**Published:** 2023-09-15

**Authors:** Vladimir Li, Youngho Lee, Chul Lee, Heebal Kim

**Affiliations:** 1 Interdisciplinary Program in Bioinformatics, Seoul National University, Seoul, Republic of Korea; 2 Laboratory of Neurogenetics of Language, The Rockefeller University, New York, NY USA; 3 Department of Agricultural Biotechnology, Research Institute for Agriculture and Life Sciences, Seoul National University, Gwanak-gu 1, Gwanak-ro, Seoul, 08826 Republic of Korea; 4 eGnome, Seoul, Republic of Korea

**Keywords:** Monkeypox, *In silico*, Drug repositioning, Molecular docking

## Abstract

**Background:**

Monkeypox is endemic to African region and has become of Global concern recently due to its outbreaks in non-endemic countries. Although, the disease was first recorded in 1970, no monkeypox specific drug or vaccine exists as of now.

**Methods:**

We applied drug repositioning method, testing effectiveness of currently approved drugs against emerging disease, as one of the most affordable approaches for discovering novel treatment measures. Techniques such as virtual ligand-based and structure-based screening were applied to identify potential drug candidates against monkeypox.

**Results:**

We narrowed down our results to 6 antiviral and 20 anti-tumor drugs that exhibit theoretically higher potency than tecovirimat, the currently approved drug for monkeypox disease.

**Conclusions:**

Our results indicated that selected drug compounds displayed strong binding affinity for p37 receptor of monkeypox virus and therefore can potentially be used in future studies to confirm their effectiveness against the disease.

**Supplementary Information:**

The online version contains supplementary material available at 10.1007/s13258-023-01449-8.

## Introduction

Monkeypox is a disease caused by monkeypox virus, an enveloped double-stranded DNA virus, which belongs to the family *Orthopoxviruses* and include vaccinia, cowpox, smallpox and variola (*Mpox (Monkeypox)*, 2023). The first human monkeypox case was identified in 1970 in the Demographic Republic of Congo after a recent eradication of smallpox in the region (Breman et al. 1980). Since then, cases of monkeypox were reported in total of 11 countries in the African continent (*Mpox (Monkeypox)*, 2023). The first monkeypox outbreak outside of Africa occurred in 2003 in the United States of America (CDC 2022). The disease gained global attention in May 2022, after multiple cases of human monkeypox were identified in non-endemic countries (*Mpox (Monkeypox)*, 2023). The natural reservoir of the virus remains unknown. According to Center for Disease Control and Prevention (CDC) monkeypox spread across the World with a total of 31,800 registered cases as of August 9, 2022 (CDC 2022). 31,425 cases occurred in countries with no previous historical reports of the virus outbreaks (CDC 2022). The virus can be transmitted through animal bites, direct contact with bodily fluids, and through skin-to-skin and intimate contacts with an infected person (CDC 2022). The major symptom of monkeypox disease is a rash on the surface of the skin; additional symptoms include fever, chills, swollen lymph nodes, weakness, muscle aches, headache and flu like symptom (CDC 2022).

p37 (encoded by F13L gene), the main extracellular protein, which is encoded by all members of the family *Orthopoxviruses*, is a 372 amino acid protein expressed during the late phase of infection. It participates in formation of the viral double membrane when the virus exits from the host cell (Blasco and Moss 1991). p37 interacts with elements of trans-Golgi (TGN) that envelope viral particles leading to formation of the triple-wrapped virus prior to being transported to cell surface and release (Blasco and Moss 1991). The protein has no analogs in the host organism making it an ideal drug target (Chen et al. 2009). Tecovirimat, also known as ST-246, was specifically developed to combat orthopoxviruses; its mechanism of action is specifically targeted towards p37, as it has been identified as the drug’s target through genetic mapping of tecovirimat-resistant mutant viruses. (Yang et al. 2005). In vivo tests demonstrated that oral administration of tecovirimat to the infected mice protected them from lethal orthopoxvirus infection (Yang et al. 2005). Study showed that p37 inhibition by tecovirimat prevented its interaction with Rab9 GTPase and TIP47, thus, halting the formation of cell-associated enveloped viruses (CEV) and extracellular enveloped viruses (EEV) (Chen et al. 2009).

Currently, no specific drug targeting monkeypox is available on the market. However, due to its genetic similarity in key regions to smallpox (96.3%), tecovirimat, a drug approved for smallpox, can be prescribed for monkepox treatment as well (Duraffour et al. 2010; Merchlinsky et al. 2019; Desai et al. 2022). Traditional methods of drug development may take decades before a successful drug candidate can be developed and delivered to patients. Drug repositioning or repurposing is a method of determining new applications of existing drugs to treat common and rare diseases (Ashburn and Thor 2004; Hurle et al. 2013; Li et al. 2016). There are several benefits involved when utilizing this method, including lower health risk, since drugs used have already been shown to be sufficiently safe, shorter time period before approving a drug for treatment and significantly less required investment (Ashburn and Thor 2004; Hurle et al. 2013; Li et al. 2016). Due to the importance of p37 protein, we selected it as a target for *in silico* drug repositioning conducted in this work.

## Materials and methods

### Data retrieval

**Fig. 1:**
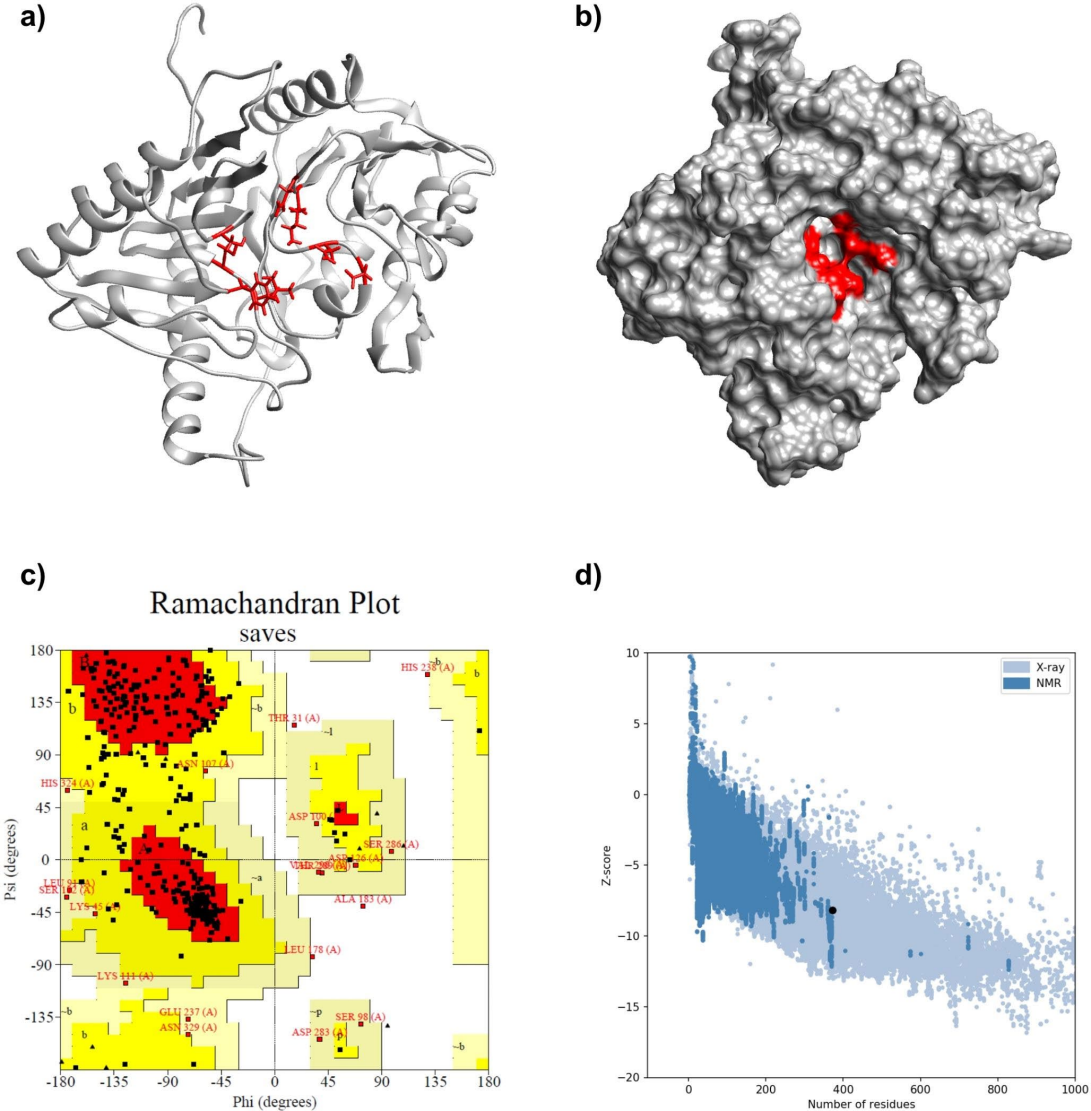
(**a**) A complete docked model of p37 protein and tecovirimat with the binding pocket (red); (**b**) A close-up view on the binding pocket of p37 with active amino acid residues (red) interacting with the ligand (cyan); (**c**) A schematic diagram of p37-tecovirimat interaction; (**d**) 3D representation of tecovirimat structure

The p37 envelope protein sequence was retrieved from National Center for Biotechnology Information (NCBI; accession number: NP_536472.1) (Sayers et al. 2022). Structure of tecovirimat in canonical simplified molecular-input line-entry system (SMILES) was retrieved from ChEMBL database (ChEMBL ID: CHEMBL1257073) (Gaulton et al. 2017). 3D structure of tecovirimat is present in Fig. 1c and d.

### 3D structures preparation

We utilized Iterative Threading ASSEmbly Refinement (I-TASSER; https://zhanggroup.org/I-TASSER/) web-server to prepare p37 protein using the template structure and determine the binding pocket for ligands (Zhang 2008; Roy et al. 2010). This server identifies suitable template found in Protein Data Bank (PDB) using threading method and then performs template-based fragment assembly of the final model(Berman et al. 2000, 2003). PROCHECK and ProSA-Web servers were utilized to assess the quality of the resulting protein model (Laskowski et al. 1993, 1996; Wiederstein and Sippl, 2007).

Open Babel software was used in this work to prepare 3D structure of ligands with added hydrogens atoms from their corresponding SMILES (O’Boyle et al. 2011).

### Virtual screening

To identify potential ligands, we performed ligand-based virtual screening (LBVS) using SWISS-SIMILARITY (SS) server of SwissDrugDesign group (http://www.swisssimilarity.ch/) and structure-based virtual screening (SBVS) using PHARMIT (https://pharmit.csb.pitt.edu/) (Sunseri and Koes 2016; Zoete et al. 2016; Bragina et al. 2022). The screened compounds from PHARMIT were retrieved using ChEMBL web-resource client (https://github.com/chembl/chembl_webresource_client) (Davies et al. 2015). LBVS allows identification of potential ligands based on their similarity with the query structure, while SBVS utilizes information of binding pocket of protein target to determine suitable candidates (Hamza et al. 2012; Li and Shah 2017).

### p37 – ligand docking

Chimera 1.15 software was used to visualize and analyze protein and ligand (Pettersen et al. 2004). Hydrogens were added to the structures and DockPrep plugin was utilized to prepare receptor structure for docking analysis (Pettersen et al. 2004; Allen et al. 2015). Protein-ligand docking was performed with AutoDock Vina software (Trott and Olson 2009). It is a molecular modelling simulation software, which docks ligands to a box, defined by the set of x, y and z coordinates (Trott and Olson 2009). AutoDock Vina is a direct successor of Autodock and is significantly faster, being able to utilize multiple cores for the simulations (Trott and Olson 2009). Box size was set to 20 for all coordinates and centered to x = 73.38, y = 69.46 and z = 64.25; the energy range was set to 3 with exhaustiveness level of 8 and 10 modes. Binding energy of the best protein-ligands modes was assessed and LigProt software was used to visualize protein-ligand interactions (Wallace et al. 1995; Laskowski and Swindells 2011). We performed p37 – tecovirimat docking and used the results as a reference for other docking simulations. LigProt software was utilized to visualize protein-ligand interaction (Supplementary Fig. 1).

## Results

### Receptor structure preparation

**Fig. 2:**
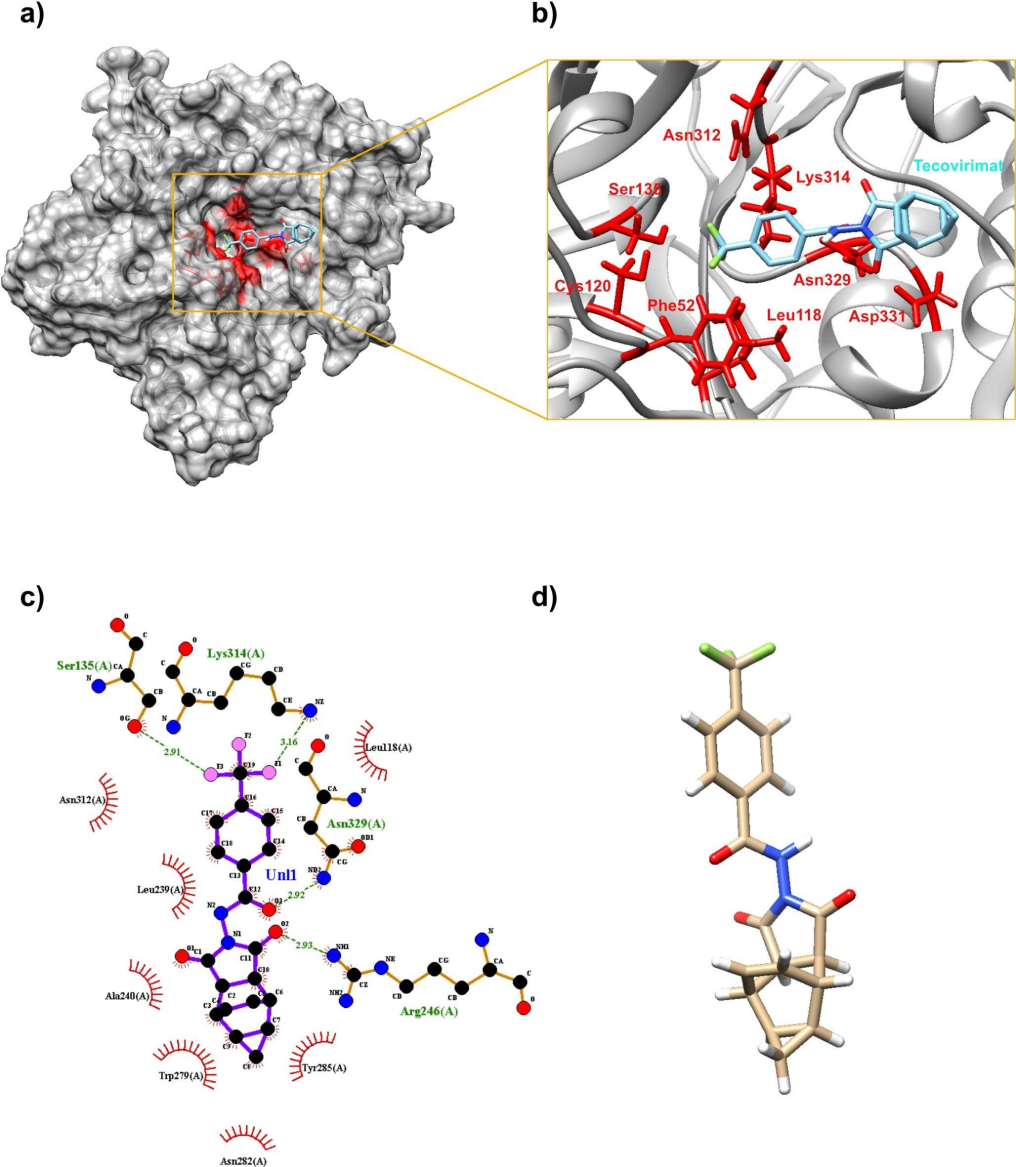
(**a**) A 3D model of p37 protein (ribbon) with highlighted active site residues (red); (**b**) A surface representation of p37 protein model with highlighted binding pocket (red); (**c**) Ramachandran plot (PROCHECK) of p37 model (233 (68.7%) residues in most favored regions, 87 (25.7%) residues in additional allowed regions, 15 (4.4%) residues in generously allowed regions, 4 (1.2%) residues in disallowed regions); (**d**) Validation of the final model with ProSA-Web (Z-score = -8.18)

Tungstate-inhibited phospholipase D (PDB: 1V0R) was selected as a template for the p37 protein. The resulting C-score predicted by I-TASSER was 0.23 with cluster size of 8, highlighting the adequate quality of this model (Fig. 2a, b). C-score is a confidence score for predicted models, it is based on the significance of threading template alignments and the convergence parameters of the structure assembly simulations (Zhang 2008; Roy et al. 2010). ProSA-Web evaluation revealed that the resulting model had a Z-score of -8.18 (Fig. 2d), which is a good indicator. Z-score is an indicator of general model quality, which represents a comparative analysis of the query model against other proteins available in PDB (Wiederstein and Sippl, 2007). PROCHECK web-server analysis indicated the following results: 233 AAs (68.7%) in the most favored regions, 87 (25.7%) AAs in additional allowed regions, 15 (4.4%) AAs in generously allowed regions and 4 (1.2%) AAs in disallowed regions (Fig. 2c). The ligand-binding site residues were predicted as follows: Phe52, Leu118, Cys120, Ser135, Asn312, Lys314, Asn329 and Asp331.

### Ligand-based virtual screening: SWISS-SIMILARITY

SWISS-SIMILARITY server allows to perform ligand-based virtual screening of chemical libraries and retrieves compounds most similar to the user’s query (Zoete et al. 2016; Bragina et al. 2022). We used tecovirimat as an input and retrieved a total of 29 approved drug compounds from ChEMBL database using variety of methods (Supplementary Table 1). The similarity of selected compounds to tecovirimat varied between 0.852 (CHEMBL3622821, upadacitinib) and 0.023 (CHEMBL480, Lansoprazole) (Supplementary able 1).

**Table 1:** The docking results of the top selected drug candidates from SWISS-SIMILARITY and Pharmit

SWISS-SIMILARITY					
#	ChEMBL ID	Drug name	Affinity (kcal/mol)	Antiviral	Antitumor
1	CHEMBL1200969	DUTASTERIDE	-9.3		
2	CHEMBL255863	NILOTINIB	-9.3		
3	CHEMBL4594271	BEROTRALSTAT	-8.6		
4	CHEMBL3813873	PEXIDARTINIB	-8.4		**α**
5	CHEMBL1422	SITAGLIPTIN	-8.2		
**Pharmit**					
**#**	**ChEMBL ID**	**Drug name**	**Affinity (kcal/mol)**	**Antiviral**	**Antitumor**
1	CHEMBL3137309	VENETOCLAX	-9.6		**α**
2	CHEMBL3414621	TAZEMETOSTAT	-9.5		**α**
3	CHEMBL1596	CARBENICILLIN INDANYL	-9.3		
4	CHEMBL282575	ESTRADIOL BENZOATE	-9.3		
5	CHEMBL3545363	GLECAPREVIR	-9.3	**α**	
6	CHEMBL2364638	UBROGEPANT	-9.2		
7	CHEMBL452231	TENIPOSIDE	-9.2		**α**
8	CHEMBL608533	MIDOSTAURIN	-9.2		**α**
9	CHEMBL3948730	UMBRALISIB	-9.1		**α**
10	CHEMBL4582651	PRALSETINIB	-9.1		**α**
11	CHEMBL1208155	ELAGOLIX	-9		
12	CHEMBL2048028	LIFITEGRAST	-9		
13	CHEMBL2178422	RIMEGEPANT	-9		
14	CHEMBL1983268	ENTRECTINIB	-8.9		**α**
15	CHEMBL2103870	LUMACAFTOR	-8.9		
16	CHEMBL3894860	TRILACICLIB	-8.9		
17	CHEMBL85	RISPERIDONE	-8.9		
18	CHEMBL1200376	BETAMETHASONE BENZOATE	-8.8		
19	CHEMBL3402762	TEPOTINIB	-8.8		**α**
20	CHEMBL3989866	BICTEGRAVIR	-8.8	**α**	
21	CHEMBL1095283	CARBENICILLIN PHENYL	-8.8		
22	CHEMBL1621	PALIPERIDONE	-8.7		
23	CHEMBL2005186	BELUMOSUDIL	-8.7		
24	CHEMBL2028663	DABRAFENIB	-8.7		**α**
25	CHEMBL3039502	DUVELISIB	-8.7		**α**
26	CHEMBL3989917	FOSNETUPITANT	-8.7		
27	CHEMBL1237021	LURASIDONE	-8.6		
28	CHEMBL3545062	VELPATASVIR	-8.6	**α**	
29	CHEMBL4297516	LURBINECTEDIN	-8.6		**α**
30	CHEMBL2105695	TELOTRISTAT ETHYL	-8.5		**α**
31	CHEMBL36506	NOVOBIOCIN	-8.5		
32	CHEMBL3989908	ENASIDENIB	-8.5		**α**
33	CHEMBL44657	ETOPOSIDE	-8.5		**α**
34	CHEMBL74632	MOXALACTAM	-8.5		
35	CHEMBL1289926	AXITINIB	-8.4		**α**
36	CHEMBL1537	AZLOCILLIN	-8.4		
37	CHEMBL2048484	CANAGLIFLOZIN	-8.4		
38	CHEMBL237500	LINAGLIPTIN	-8.4		
39	CHEMBL3544914	TEZACAFTOR	-8.4		
40	CHEMBL408	TROGLITAZONE	-8.4		
41	CHEMBL603	ZAFIRLUKAST	-8.4		
42	CHEMBL1200645	ETOPOSIDE PHOSPHATE	-8.3		**α**
43	CHEMBL1201204	CEFPIRAMIDE	-8.3		
44	CHEMBL13828	OXATOMIDE	-8.3		
45	CHEMBL1731	MEZLOCILLIN	-8.3		
46	CHEMBL222645	FLOXACILLIN	-8.3		
47	CHEMBL2216870	IDELALISIB	-8.3		**α**
48	CHEMBL3989958	IVOSIDENIB	-8.3		**α**
49	CHEMBL4065616	REMDESIVIR	-8.3	**α**	
50	CHEMBL1095777	INDACATEROL	-8.2		
51	CHEMBL1200430	ESTRADIOL ACETATE	-8.2		
52	CHEMBL1229211	DOLUTEGRAVIR	-8.2	**α**	
53	CHEMBL2403238	CABOTEGRAVIR	-8.2	**α**	
54	CHEMBL3188267	CAPMATINIB	-8.2		**α**
55	CHEMBL4297528	RISDIPLAM	-8.2		
56	CHEMBL512351	BETRIXABAN	-8.2		

### Structure-based virtual screening: PHARMIT

PHARMIT is a web-server designed to perform structure-based virtual screening using pharmacophore, molecular shape and energy minimization (Sunseri and Koes 2016). We utilized exclusive shape with tolerance 1.0 and selected pharmacophore parameters were set with radius of 1. As with LBVS method, we screened ChEMBL30 database and screened a total of 11,612,000 compounds and only phase 4 drugs were retrieved for the final analysis. As a result, a total of 592 phase 4 drugs were selected for the docking analysis (Supplementary Table 2).

### Receptor – ligand docking

The p37 – tecovirimat docking demonstrated strong binding with the top model scoring at -8.2 kcal/mol, which was subsequently used as a reference point for further analysis (Fig. 1a, b. The complete list of docking results for ligands retrieved from ligand screening of SWISS-SIMILARITY and PHARMIT can be found in Supplementary Tables 1 and Supplementary Table 2 correspondingly. The top scoring ligand obtained via SWISS-SIMILARITY was durasteride, an antiandrogenic compound used to treat symptomatic benign prostatic hyperplasia, with − 9.3 kcal/mol binding energy and the lowest scoring ligand was sitagliptin, a dipeptidyl peptidase-4 (DPP-4) inhibitor used to manage type 2 diabetes melitus, with − 8.2 kcal/mol binding energy score (Wishart et al. 2018). The docking analysis of 592 phase 4 drugs screened with the help of PHARMIT produced multiple results (56 drug compounds) with top scoring models ranging from − 9.6 - -8.2 kcal/mol binding energy (Table 1). Among selected drugs from PHARMIT screening six compounds were identified as antiviral drugs including glecaprevir (-9.3 kcal/mol), bictegravir (-8.8 kcal/mol), velpatasvir (-8.6 kcal/mol), remdesivir (-8.3 kcal/mol), dolutegravir (-8.2 kcal/mol) and cabotegravir (-8.2 kcal/mol) (Table 1). 19 compounds were identified as various anticancer drugs (Table 1). Screening results of SWISS-SIMILARITY revealed no antiviral drugs and one antitumor agent (pexidartinib, -8.5 kcal/mol) (Table 1) (Wishart et al. 2018).

## Discussion

Currently there are no monkeypox specific vaccines and drugs available and smallpox treatment is prescribed to counteract monkeypox disease (Breman et al. 1980; CDC 2022; *Mpox (Monkeypox)*, 2023). Tecovirimat is one of the drugs prescribed to treat smallpox incidence, which demonstrated decent effectiveness (Duraffour et al. 2010; Desai et al. 2022). It was discovered through high-throughput screening in 2002 and was shown to be effective against all orthopoxviruses (Duraffour et al. 2010; Merchlinsky et al. 2019; Desai et al. 2022). In 2018, the Food and Drug Administration (FDA) approved the drug for smallpox treatment (Hoy 2018). Tecovirimat inhibits the formation of p37, a major envelope protein, required to produce virions (Duraffour et al. 2010). Thus, it limits the virus from exiting infected cells preventing spread of the infection (Duraffour et al. 2010; Desai et al. 2022).

In this study we attempted to perform virtual screening based on the tecovirimat mode of binding to p37 envelope protein utilizing both LBVS and SBVS methods. To our knowledge this is the first drug repositioning study dedicated to identify monkeypox drug candidates using both virtual screening methods. We, first, prepared tertiary structure of p37 protein using I-TASSER server and analyzed it using PROCHECK (Ramachandran plot) and PROSA-Web server. Overall, the model demonstrated good ratio of residues in allowed and disallowed regions with strong Z-score (X-ray, NMR) in the range normally found in native proteins of the similar size, indicating a suitability of the predicted tertiary structure for further analysis. The ligand-binding pocket was also predicted by I-TASSER server and included a total of eight residues.

p37–tecovirimat docking was performed to create a reference result with the strongest binding energy of -8.2 kcal/mol. We considered this value as the lowest threshold for the screening drugs to be considered as candidates for monkeypox treatment therapy. SWISS-SIMILARITY server was used to perform LBVS and PHARMIT was utilized for SBVS. The important aspect of any drug discovery process is to assume its safety to the user. After primary screening (LBVS and SBVS) we selected only phase 4 drugs (approved for use in the general population). Since monkeypox is a viral disease, we were primarily interested in antiviral drugs. We also included anti-tumor drugs in our list since previous repurposing studies showed successful repositioning of the said compounds to treat or alleviate symptoms of viral diseases (Ciliberto et al. 2020; Aldea et al. 2021). LBVS produced only a single antitumor drug, while SBVS identified six antiviral agents and 19 antitumor compounds (Table 1). LBVS and SBVS produced significantly different results due to the totally opposite methods utilized in drug screening. Since LBVS was focused on the ligand structure, it identified compounds that closely resembled tecovirimat, whereas SBVS which is based on the binding pocket of the receptor, determined drugs that would most likely fit in it. Thus, the drugs produced by LBVS and SBVS and selected for the final docking did not overlap as we originally theorized. However, due to this disagreement, we were able to obtain higher variety in the compounds to analyze and compare.

The final candidates were selected based on their interaction strength with p37, mimicking possible real receptor-ligand interactions. Our results indicate the possibility of identified candidate drugs as treatments against monkeypox. We believe that results produced in this study will bring us one step closer to identifying a more effective drug in treating and alleviating symptoms of monkeypox. Nevertheless, one should consider that our results are a product of computer simulations and to produce actual viable data, *in vitro/in vivo* confirmations are required to confirm the effectiveness of the drugs, which will be performed in future studies.

## Conclusions

Developing novel drugs against emerging diseases such as monkeypox takes significant amount of time and resources. Alternatively, drug repurposing provides a relatively quick and efficient approach to identify drug candidates from existing drug pool. Thus, this study was conducted to identify those candidates using SBVS and LBVS methods together with 3D protein structure modelling and virtual docking. The results revealed 6 antiviral and 20 anti-tumor drugs with binding affinity stronger than that of tecovirimat, indicating that the selected compounds have high chances to be effective against the disease.

### Electronic supplementary material

Below is the link to the electronic supplementary material.

Supplementary Material 1

## Data Availability

The datasets used in this study are available in the ChEMBL database (https://www.ebi.ac.uk/chembl/) and all accession numbers are provided in the article. The additional information about this work is available from the corresponding author upon a reasonable request.
