# Supplementary material for: Repurposing existing drugs for monkeypox: applications of virtual screening methods
Source: Genes Genomics. 2023 Sep 15;45(11):1347–55. doi: 10.1007/s13258-023-01449-8 (PMC10587275; doi:10.1007/s13258-023-01449-8)
Supplement: Supplementary file 1 — Supplementary Material 1 [file 13258_2023_1449_MOESM1_ESM.docx]

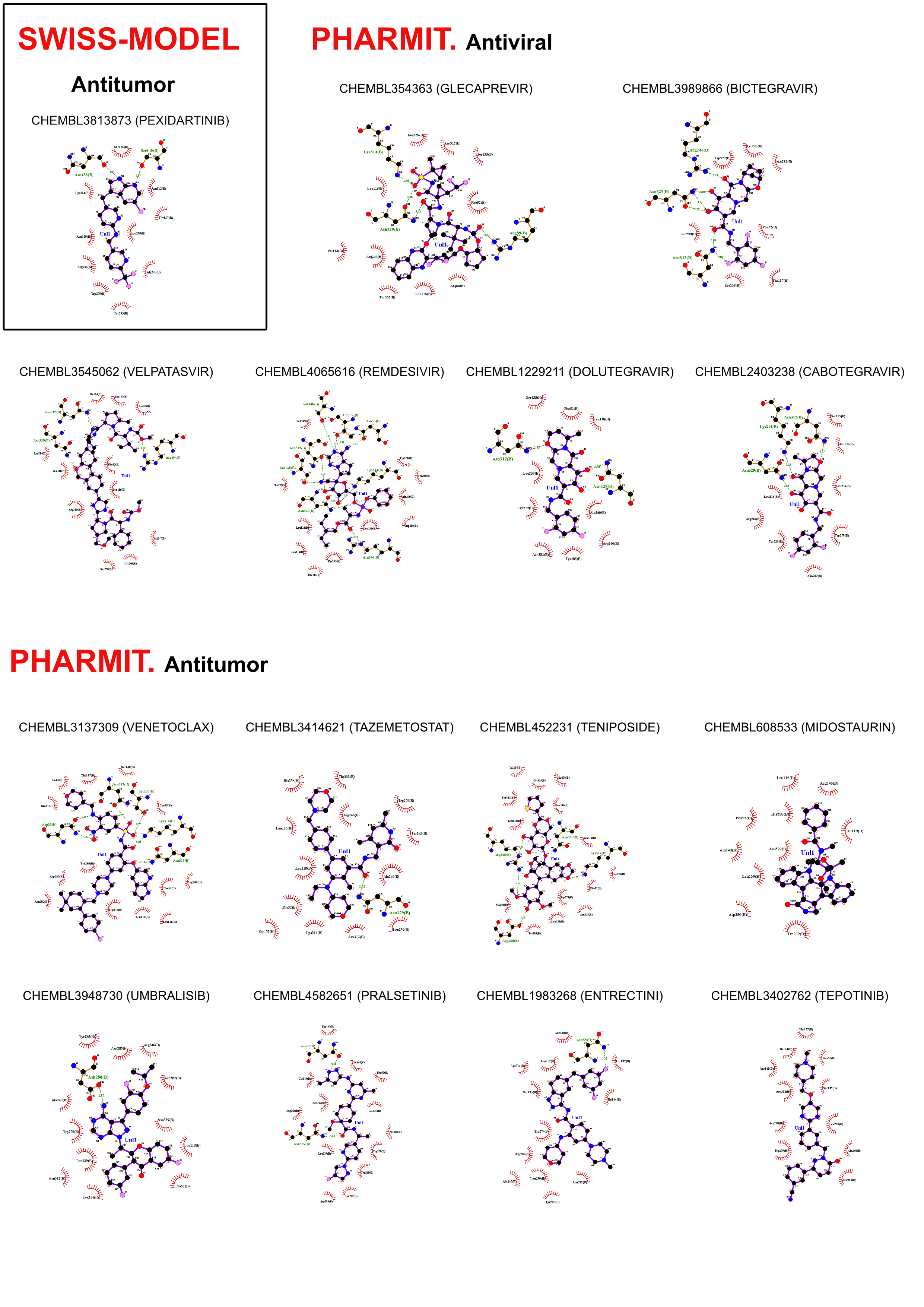


**Supplementary figure 1-1.** Docking interactions between P37 and the selected drug candidates.


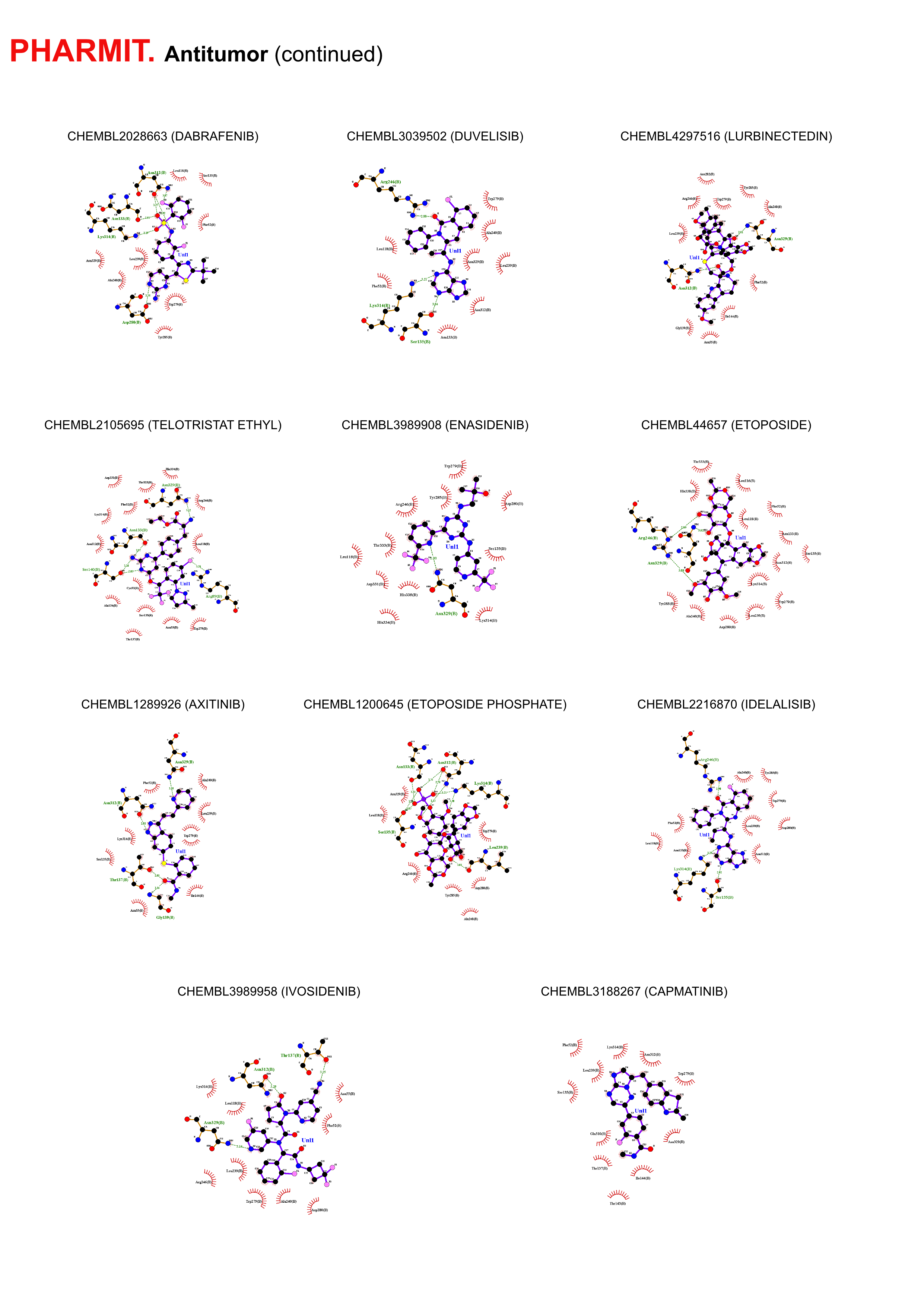


**Supplementary figure 1-2.** Docking interactions between P37 and the selected drug candidates.

**Supplementary table 1**. A complete list of P37-ligand docking results of ligands screened with SWISS-SIMILARITY.

| **#** | **ChEMBL ID** | **Drug name** | **Mwt** | **Alogp** | **Score** | **Affinity (kcal/mol)** |
| --- | --- | --- | --- | --- | --- | --- |
| 1 | CHEMBL1200969 | DUTASTERIDE | 528.54 | 6.58 | 0.297 | -9.3 |
| 2 | CHEMBL255863 | NILOTINIB | 529.53 | 6.36 | 0.049 | -9.3 |
| 3 | CHEMBL4594271 | BEROTRALSTAT | 562.57 | 5.7 | 0.077 | -8.6 |
| 4 | CHEMBL3813873 | PEXIDARTINIB | 417.82 | 5.23 | 0.027 | -8.4 |
| 5 | CHEMBL1422 | SITAGLIPTIN | 407.32 | 2.02 | 0.084 | -8.2 |
| 6 | CHEMBL231068 | FLIBANSERIN | 390.41 | 3.17 | 0.845 | -7.9 |
| 7 | CHEMBL3183409 | APALUTAMIDE | 477.44 | 3.53 | 0.074 | -7.8 |
| 8 | CHEMBL206253 | NETUPITANT | 578.6 | 6.79 | 0.101 | -7.7 |
| 9 | CHEMBL118 | CELECOXIB | 381.38 | 3.51 | 0.052 | -7.7 |
| 10 | CHEMBL354541 | LOMITAPIDE | 693.73 | 8.38 | 0.25 | -7.6 |
| 11 | CHEMBL3545185 | SELINEXOR | 443.31 | 3.39 | 0.045 | -7.5 |
| 12 | CHEMBL2364608 | DORAVIRINE | 425.75 | 2.65 | 0.032 | -7.5 |
| 13 | CHEMBL1201863 | DEXLANSOPRAZOLE | 369.37 | 3.52 | 0.031 | -7.5 |
| 14 | CHEMBL480 | LANSOPRAZOLE | 369.37 | 3.52 | 0.023 | -7.5 |
| 15 | CHEMBL1684 | BENDROFLUMETHIAZIDE | 421.42 | 1.63 | 0.026 | -7.4 |
| 16 | CHEMBL3707331 | ROLAPITANT | 500.48 | 5.73 | 0.175 | -7.2 |
| 17 | CHEMBL1082407 | ENZALUTAMIDE | 464.44 | 3.99 | 0.086 | -7.1 |
| 18 | CHEMBL652 | FLECAINIDE | 414.35 | 3.44 | 0.165 | -7 |
| 19 | CHEMBL298470 | TAFENOQUINE | 463.5 | 5.91 | 0.082 | -7 |
| 20 | CHEMBL3622821 | UPADACITINIB | 380.37 | 2.91 | 0.852 | -6.9 |
| 21 | CHEMBL1274 | NILUTAMIDE | 317.22 | 2.45 | 0.104 | -6.9 |
| 22 | CHEMBL960 | LEFLUNOMIDE | 270.21 | 3.25 | 0.281 | -6.8 |
| 23 | CHEMBL422 | TRIFLUOPERAZINE | 407.51 | 4.95 | 0.054 | -6.8 |
| 24 | CHEMBL1200472 | QUAZEPAM | 386.8 | 5.03 | 0.025 | -6.7 |
| 25 | CHEMBL970 | HALAZEPAM | 352.74 | 4.09 | 0.031 | -6.6 |
| 26 | CHEMBL41 | FLUOXETINE | 309.33 | 4.44 | 0.052 | -6.5 |
| 27 | CHEMBL400599 | BENFLUOREX | 351.37 | 4.08 | 0.063 | -6.4 |
| 28 | CHEMBL806 | FLUTAMIDE | 276.21 | 3.21 | 0.08 | -6.2 |
| 29 | CHEMBL973 | TERIFLUNOMIDE | 270.21 | 3 | 0.266 | -6.1 |

**Supplementary table 2**. A complete list of P37-ligand docking results of ligands screened with Pharmit.

| **#** | **ChEMBL ID** | **Drug name** | **Affinity (kcal/mol)** |
| --- | --- | --- | --- |
| 1 | CHEMBL3137309 | VENETOCLAX | -9.6 |
| 2 | CHEMBL3414621 | TAZEMETOSTAT | -9.5 |
| 3 | CHEMBL1596 | CARBENICILLIN INDANYL | -9.3 |
| 4 | CHEMBL282575 | ESTRADIOL BENZOATE | -9.3 |
| 5 | CHEMBL3545363 | GLECAPREVIR | -9.3 |
| 6 | CHEMBL2364638 | UBROGEPANT | -9.2 |
| 7 | CHEMBL452231 | TENIPOSIDE | -9.2 |
| 8 | CHEMBL608533 | MIDOSTAURIN | -9.2 |
| 9 | CHEMBL3948730 | UMBRALISIB | -9.1 |
| 10 | CHEMBL4582651 | PRALSETINIB | -9.1 |
| 11 | CHEMBL1208155 | ELAGOLIX | -9 |
| 12 | CHEMBL2048028 | LIFITEGRAST | -9 |
| 13 | CHEMBL2178422 | RIMEGEPANT | -9 |
| 14 | CHEMBL1983268 | ENTRECTINIB | -8.9 |
| 15 | CHEMBL2103870 | LUMACAFTOR | -8.9 |
| 16 | CHEMBL3894860 | TRILACICLIB | -8.9 |
| 17 | CHEMBL85 | RISPERIDONE | -8.9 |
| 18 | CHEMBL1095283 | CARBENICILLIN PHENYL | -8.8 |
| 19 | CHEMBL1200376 | BETAMETHASONE BENZOATE | -8.8 |
| 20 | CHEMBL3402762 | TEPOTINIB | -8.8 |
| 21 | CHEMBL3989866 | BICTEGRAVIR | -8.8 |
| 22 | CHEMBL1621 | PALIPERIDONE | -8.7 |
| 23 | CHEMBL2005186 | BELUMOSUDIL | -8.7 |
| 24 | CHEMBL2028663 | DABRAFENIB | -8.7 |
| 25 | CHEMBL3039502 | DUVELISIB | -8.7 |
| 26 | CHEMBL3989917 | FOSNETUPITANT | -8.7 |
| 27 | CHEMBL1237021 | LURASIDONE | -8.6 |
| 28 | CHEMBL3545062 | VELPATASVIR | -8.6 |
| 29 | CHEMBL4297516 | LURBINECTEDIN | -8.6 |
| 30 | CHEMBL2105695 | TELOTRISTAT ETHYL | -8.5 |
| 31 | CHEMBL36506 | NOVOBIOCIN | -8.5 |
| 32 | CHEMBL3989908 | ENASIDENIB | -8.5 |
| 33 | CHEMBL44657 | ETOPOSIDE | -8.5 |
| 34 | CHEMBL74632 | MOXALACTAM | -8.5 |
| 35 | CHEMBL1289926 | AXITINIB | -8.4 |
| 36 | CHEMBL1537 | AZLOCILLIN | -8.4 |
| 37 | CHEMBL2048484 | CANAGLIFLOZIN | -8.4 |
| 38 | CHEMBL237500 | LINAGLIPTIN | -8.4 |
| 39 | CHEMBL3544914 | TEZACAFTOR | -8.4 |
| 40 | CHEMBL408 | TROGLITAZONE | -8.4 |
| 41 | CHEMBL603 | ZAFIRLUKAST | -8.4 |
| 42 | CHEMBL1200645 | ETOPOSIDE PHOSPHATE | -8.3 |
| 43 | CHEMBL1201204 | CEFPIRAMIDE | -8.3 |
| 44 | CHEMBL13828 | OXATOMIDE | -8.3 |
| 45 | CHEMBL1731 | MEZLOCILLIN | -8.3 |
| 46 | CHEMBL2216870 | IDELALISIB | -8.3 |
| 47 | CHEMBL222645 | FLOXACILLIN | -8.3 |
| 48 | CHEMBL3989958 | IVOSIDENIB | -8.3 |
| 49 | CHEMBL4065616 | REMDESIVIR | -8.3 |
| 50 | CHEMBL1095777 | INDACATEROL | -8.2 |
| 51 | CHEMBL1200430 | ESTRADIOL ACETATE | -8.2 |
| 52 | CHEMBL1229211 | DOLUTEGRAVIR | -8.2 |
| 53 | CHEMBL2403238 | CABOTEGRAVIR | -8.2 |
| 54 | CHEMBL3188267 | CAPMATINIB | -8.2 |
| 55 | CHEMBL4297528 | RISDIPLAM | -8.2 |
| 56 | CHEMBL512351 | BETRIXABAN | -8.2 |
| 57 | CHEMBL1017 | TELMISARTAN | -8.1 |
| 58 | CHEMBL1117 | IDARUBICIN | -8.1 |
| 59 | CHEMBL1138 | EZETIMIBE | -8.1 |
| 60 | CHEMBL1232801 | FOLINIC ACID | -8.1 |
| 61 | CHEMBL1289494 | TIVOZANIB | -8.1 |
| 62 | CHEMBL1372950 | NICERGOLINE | -8.1 |
| 63 | CHEMBL1601 | CEFONICID | -8.1 |
| 64 | CHEMBL2103875 | TRAMETINIB | -8.1 |
| 65 | CHEMBL2135460 | TERLIPRESSIN | -8.1 |
| 66 | CHEMBL3218576 | COPANLISIB | -8.1 |
| 67 | CHEMBL3301594 | FOSTEMSAVIR | -8.1 |
| 68 | CHEMBL3707313 | TRIFAROTENE | -8.1 |
| 69 | CHEMBL4559134 | SELPERCATINIB | -8.1 |
| 70 | CHEMBL472 | GLYBURIDE | -8.1 |
| 71 | CHEMBL550348 | DEFERASIROX | -8.1 |
| 72 | CHEMBL957 | BOSENTAN | -8.1 |
| 73 | CHEMBL1096 | AMLEXANOX | -8 |
| 74 | CHEMBL1201746 | PRALATREXATE | -8 |
| 75 | CHEMBL1481 | GLIMEPIRIDE | -8 |
| 76 | CHEMBL1487 | ATORVASTATIN | -8 |
| 77 | CHEMBL1908841 | LEVOLEUCOVORIN | -8 |
| 78 | CHEMBL1909289 | XENAZOIC ACID | -8 |
| 79 | CHEMBL1963681 | AVANAFIL | -8 |
| 80 | CHEMBL206253 | NETUPITANT | -8 |
| 81 | CHEMBL2103830 | FOSTAMATINIB | -8 |
| 82 | CHEMBL231068 | FLIBANSERIN | -8 |
| 83 | CHEMBL297302 | BENPERIDOL | -8 |
| 84 | CHEMBL3545185 | SELINEXOR | -8 |
| 85 | CHEMBL115 | INDINAVIR | -7.9 |
| 86 | CHEMBL1200690 | LYPRESSIN | -7.9 |
| 87 | CHEMBL1259059 | SOFOSBUVIR | -7.9 |
| 88 | CHEMBL1306 | TERCONAZOLE | -7.9 |
| 89 | CHEMBL1359 | ERTAPENEM | -7.9 |
| 90 | CHEMBL161 | CEFTRIAXONE | -7.9 |
| 91 | CHEMBL2103855 | TELOTRISTAT | -7.9 |
| 92 | CHEMBL254316 | RALTEGRAVIR | -7.9 |
| 93 | CHEMBL3286830 | LORLATINIB | -7.9 |
| 94 | CHEMBL3707372 | VOXILAPREVIR | -7.9 |
| 95 | CHEMBL92870 | LIDOFLAZINE | -7.9 |
| 96 | CHEMBL1108 | DROPERIDOL | -7.8 |
| 97 | CHEMBL1146 | CEFAMANDOLE | -7.8 |
| 98 | CHEMBL1173655 | AFATINIB | -7.8 |
| 99 | CHEMBL1183349 | ISAVUCONAZONIUM | -7.8 |
| 100 | CHEMBL1201116 | HETACILLIN | -7.8 |
| 101 | CHEMBL1251 | GANIRELIX ACETATE | -7.8 |
| 102 | CHEMBL1421 | DASATINIB | -7.8 |
| 103 | CHEMBL1622 | FOLIC ACID | -7.8 |
| 104 | CHEMBL1697844 | NICOFURANOSE | -7.8 |
| 105 | CHEMBL2103929 | LYMECYCLINE | -7.8 |
| 106 | CHEMBL2107830 | EMPAGLIFLOZIN | -7.8 |
| 107 | CHEMBL218650 | DELAMANID | -7.8 |
| 108 | CHEMBL225072 | PEMETREXED | -7.8 |
| 109 | CHEMBL238071 | VINDESINE | -7.8 |
| 110 | CHEMBL270190 | ALVIMOPAN | -7.8 |
| 111 | CHEMBL282724 | SITAXENTAN | -7.8 |
| 112 | CHEMBL3351077 | CEFSULODIN | -7.8 |
| 113 | CHEMBL3889654 | LAROTRECTINIB | -7.8 |
| 114 | CHEMBL421 | SULFASALAZINE | -7.8 |
| 115 | CHEMBL4535757 | SOTORASIB | -7.8 |
| 116 | CHEMBL4594250 | NETARSUDIL | -7.8 |
| 117 | CHEMBL685 | MEBENDAZOLE | -7.8 |
| 118 | CHEMBL1200368 | BENTIROMIDE | -7.7 |
| 119 | CHEMBL1229517 | VEMURAFENIB | -7.7 |
| 120 | CHEMBL14376 | ILOPERIDONE | -7.7 |
| 121 | CHEMBL1443 | NAFCILLIN | -7.7 |
| 122 | CHEMBL1520 | VARDENAFIL | -7.7 |
| 123 | CHEMBL157101 | KETOCONAZOLE | -7.7 |
| 124 | CHEMBL1741134 | PICOSULFURIC ACID | -7.7 |
| 125 | CHEMBL1742423 | BILASTINE | -7.7 |
| 126 | CHEMBL2364632 | SARECYCLINE | -7.7 |
| 127 | CHEMBL2396661 | ALPELISIB | -7.7 |
| 128 | CHEMBL3039520 | LASMIDITAN | -7.7 |
| 129 | CHEMBL3301612 | ENCORAFENIB | -7.7 |
| 130 | CHEMBL3545367 | LEMBOREXANT | -7.7 |
| 131 | CHEMBL3989915 | DIFELIKEFALIN | -7.7 |
| 132 | CHEMBL4066936 | VERICIGUAT | -7.7 |
| 133 | CHEMBL41286 | DIACEREIN | -7.7 |
| 134 | CHEMBL1200692 | OLMESARTAN MEDOXOMIL | -7.6 |
| 135 | CHEMBL1201046 | CEFORANIDE | -7.6 |
| 136 | CHEMBL1201304 | INDOCYANINE GREEN | -7.6 |
| 137 | CHEMBL121 | ROSIGLITAZONE | -7.6 |
| 138 | CHEMBL1287853 | FEDRATINIB | -7.6 |
| 139 | CHEMBL1450 | ATOVAQUONE | -7.6 |
| 140 | CHEMBL17157 | TERFENADINE | -7.6 |
| 141 | CHEMBL189963 | PALBOCICLIB | -7.6 |
| 142 | CHEMBL2303613 | CEFODIZIME | -7.6 |
| 143 | CHEMBL4297513 | IBREXAFUNGERP | -7.6 |
| 144 | CHEMBL4298128 | ELEXACAFTOR | -7.6 |
| 145 | CHEMBL46286 | OMACETAXINE MEPESUCCINATE | -7.6 |
| 146 | CHEMBL601719 | CRIZOTINIB | -7.6 |
| 147 | CHEMBL927 | CEFDINIR | -7.6 |
| 148 | CHEMBL1201760 | BESIFLOXACIN | -7.5 |
| 149 | CHEMBL1201863 | DEXLANSOPRAZOLE | -7.5 |
| 150 | CHEMBL1214124 | PERAMPANEL | -7.5 |
| 151 | CHEMBL1257015 | PICOTAMIDE | -7.5 |
| 152 | CHEMBL1328 | PENTAGASTRIN | -7.5 |
| 153 | CHEMBL1397 | POSACONAZOLE | -7.5 |
| 154 | CHEMBL1404 | RANOLAZINE | -7.5 |
| 155 | CHEMBL1466 | DICUMAROL | -7.5 |
| 156 | CHEMBL1471 | APREPITANT | -7.5 |
| 157 | CHEMBL1770248 | ERTUGLIFLOZIN | -7.5 |
| 158 | CHEMBL1909288 | TRIACETYLDIPHENOLISATIN | -7.5 |
| 159 | CHEMBL198362 | RIVAROXABAN | -7.5 |
| 160 | CHEMBL2107011 | OXYPERTINE | -7.5 |
| 161 | CHEMBL2107825 | TENOFOVIR ALAFENAMIDE | -7.5 |
| 162 | CHEMBL227875 | PRETOMANID | -7.5 |
| 163 | CHEMBL231813 | TELAPREVIR | -7.5 |
| 164 | CHEMBL2364608 | DORAVIRINE | -7.5 |
| 165 | CHEMBL345524 | ANTRAFENINE | -7.5 |
| 166 | CHEMBL3545110 | RIBOCICLIB | -7.5 |
| 167 | CHEMBL451887 | CARFILZOMIB | -7.5 |
| 168 | CHEMBL480 | LANSOPRAZOLE | -7.5 |
| 169 | CHEMBL528 | CEFTIZOXIME | -7.5 |
| 170 | CHEMBL729 | LOPINAVIR | -7.5 |
| 171 | CHEMBL799 | CILOSTAZOL | -7.5 |
| 172 | CHEMBL893 | DICLOXACILLIN | -7.5 |
| 173 | CHEMBL939 | GEFITINIB | -7.5 |
| 174 | CHEMBL1096885 | VALRUBICIN | -7.4 |
| 175 | CHEMBL135400 | ZOPICLONE | -7.4 |
| 176 | CHEMBL1522 | ESZOPICLONE | -7.4 |
| 177 | CHEMBL1524273 | PHTHALYLSULFATHIAZOLE | -7.4 |
| 178 | CHEMBL159 | VINBLASTINE | -7.4 |
| 179 | CHEMBL1888176 | TROSPIUM | -7.4 |
| 180 | CHEMBL1908370 | FINAFLOXACIN | -7.4 |
| 181 | CHEMBL2010601 | IVACAFTOR | -7.4 |
| 182 | CHEMBL24828 | VANDETANIB | -7.4 |
| 183 | CHEMBL277522 | FENBUFEN | -7.4 |
| 184 | CHEMBL398435 | TICAGRELOR | -7.4 |
| 185 | CHEMBL4297185 | DAROLUTAMIDE | -7.4 |
| 186 | CHEMBL437765 | RIFAMYCIN | -7.4 |
| 187 | CHEMBL454446 | CEFDITOREN PIVOXIL | -7.4 |
| 188 | CHEMBL61 | PODOFILOX | -7.4 |
| 189 | CHEMBL1201039 | BENZTHIAZIDE | -7.3 |
| 190 | CHEMBL1201087 | CABERGOLINE | -7.3 |
| 191 | CHEMBL1206690 | PARECOXIB | -7.3 |
| 192 | CHEMBL126 | LINEZOLID | -7.3 |
| 193 | CHEMBL1272 | REPAGLINIDE | -7.3 |
| 194 | CHEMBL1519 | TRANDOLAPRIL | -7.3 |
| 195 | CHEMBL158 | AZTREONAM | -7.3 |
| 196 | CHEMBL1684 | BENDROFLUMETHIAZIDE | -7.3 |
| 197 | CHEMBL189171 | ACEMETACIN | -7.3 |
| 198 | CHEMBL191 | LOSARTAN | -7.3 |
| 199 | CHEMBL529888 | GLIBORNURIDE | -7.3 |
| 200 | CHEMBL568 | OXAZEPAM | -7.3 |
| 201 | CHEMBL64391 | ITRACONAZOLE | -7.3 |
| 202 | CHEMBL1013 | LORACARBEF | -7.2 |
| 203 | CHEMBL107 | COLCHICINE | -7.2 |
| 204 | CHEMBL1200971 | CEPHALOGLYCIN | -7.2 |
| 205 | CHEMBL1214 | CARBENICILLIN | -7.2 |
| 206 | CHEMBL1289601 | LENVATINIB | -7.2 |
| 207 | CHEMBL1324 | TOLCAPONE | -7.2 |
| 208 | CHEMBL15770 | SULINDAC | -7.2 |
| 209 | CHEMBL1730 | CEFOTAXIME | -7.2 |
| 210 | CHEMBL2105224 | PHOLCODINE | -7.2 |
| 211 | CHEMBL2105637 | DELAFLOXACIN | -7.2 |
| 212 | CHEMBL244888 | NIFUROXAZIDE | -7.2 |
| 213 | CHEMBL3137301 | SACUBITRIL | -7.2 |
| 214 | CHEMBL3182343 | PIVAMPICILLIN | -7.2 |
| 215 | CHEMBL396778 | SAFINAMIDE | -7.2 |
| 216 | CHEMBL468 | THALIDOMIDE | -7.2 |
| 217 | CHEMBL514800 | APREMILAST | -7.2 |
| 218 | CHEMBL580 | LORAZEPAM | -7.2 |
| 219 | CHEMBL646 | TRIAZOLAM | -7.2 |
| 220 | CHEMBL746 | NEDOCROMIL | -7.2 |
| 221 | CHEMBL1186579 | METHYLNALTREXONE | -7.1 |
| 222 | CHEMBL1201336 | FOSPHENYTOIN | -7.1 |
| 223 | CHEMBL1256786 | FORMOTEROL | -7.1 |
| 224 | CHEMBL1496 | ROSUVASTATIN | -7.1 |
| 225 | CHEMBL1502 | PANTOPRAZOLE | -7.1 |
| 226 | CHEMBL1589 | ACETOHEXAMIDE | -7.1 |
| 227 | CHEMBL1614701 | SELUMETINIB | -7.1 |
| 228 | CHEMBL1664 | FOSAMPRENAVIR | -7.1 |
| 229 | CHEMBL1909072 | PIPAMAZINE | -7.1 |
| 230 | CHEMBL2107831 | LUSUTROMBOPAG | -7.1 |
| 231 | CHEMBL2218896 | NABILONE | -7.1 |
| 232 | CHEMBL24778 | SILODOSIN | -7.1 |
| 233 | CHEMBL291157 | ROSOXACIN | -7.1 |
| 234 | CHEMBL53292 | ISOXICAM | -7.1 |
| 235 | CHEMBL575 | METHICILLIN | -7.1 |
| 236 | CHEMBL585 | TRIAMTERENE | -7.1 |
| 237 | CHEMBL661 | ALPRAZOLAM | -7.1 |
| 238 | CHEMBL1075 | MORICIZINE | -7 |
| 239 | CHEMBL1089221 | BENDAZAC | -7 |
| 240 | CHEMBL1110 | ALOSETRON | -7 |
| 241 | CHEMBL119443 | ERGONOVINE | -7 |
| 242 | CHEMBL141305 | CYCLOFENIL | -7 |
| 243 | CHEMBL1436 | CEFUROXIME | -7 |
| 244 | CHEMBL1464 | WARFARIN | -7 |
| 245 | CHEMBL1480987 | CYCLANDELATE | -7 |
| 246 | CHEMBL1697737 | CLOTIAZEPAM | -7 |
| 247 | CHEMBL2103772 | RACECADOTRIL | -7 |
| 248 | CHEMBL308954 | ETRAVIRINE | -7 |
| 249 | CHEMBL317094 | IMIDAPRIL | -7 |
| 250 | CHEMBL3187723 | BINIMETINIB | -7 |
| 251 | CHEMBL329522 | EXIFONE | -7 |
| 252 | CHEMBL4 | OFLOXACIN | -7 |
| 253 | CHEMBL409 | BICALUTAMIDE | -7 |
| 254 | CHEMBL427216 | GLICLAZIDE | -7 |
| 255 | CHEMBL832 | SULFINPYRAZONE | -7 |
| 256 | CHEMBL850 | SPARFLOXACIN | -7 |
| 257 | CHEMBL898 | DIFLUNISAL | -7 |
| 258 | CHEMBL981 | FENOFIBRIC ACID | -7 |
| 259 | CHEMBL996 | CEFOXITIN | -7 |
| 260 | CHEMBL1000 | CETIRIZINE | -6.9 |
| 261 | CHEMBL101 | PHENYLBUTAZONE | -6.9 |
| 262 | CHEMBL1071 | OXAPROZIN | -6.9 |
| 263 | CHEMBL1095930 | CEFUROXIME AXETIL | -6.9 |
| 264 | CHEMBL1096882 | FLUDARABINE PHOSPHATE | -6.9 |
| 265 | CHEMBL1111 | AMBRISENTAN | -6.9 |
| 266 | CHEMBL1168 | RAMIPRIL | -6.9 |
| 267 | CHEMBL117785 | TETRABENAZINE | -6.9 |
| 268 | CHEMBL1189679 | PALONOSETRON | -6.9 |
| 269 | CHEMBL1201016 | CEFPODOXIME PROXETIL | -6.9 |
| 270 | CHEMBL1201314 | VALGANCICLOVIR | -6.9 |
| 271 | CHEMBL1256841 | NIALAMIDE | -6.9 |
| 272 | CHEMBL13280 | FLUNITRAZEPAM | -6.9 |
| 273 | CHEMBL1401 | NITAZOXANIDE | -6.9 |
| 274 | CHEMBL146095 | GLAFENINE | -6.9 |
| 275 | CHEMBL1541 | CEFIXIME | -6.9 |
| 276 | CHEMBL1644 | CEFADROXIL | -6.9 |
| 277 | CHEMBL2105760 | BREXPIPRAZOLE | -6.9 |
| 278 | CHEMBL2106794 | NAFTAZONE | -6.9 |
| 279 | CHEMBL2106915 | METHOPHOLINE | -6.9 |
| 280 | CHEMBL264374 | BEZAFIBRATE | -6.9 |
| 281 | CHEMBL3353410 | OSIMERTINIB | -6.9 |
| 282 | CHEMBL3622821 | UPADACITINIB | -6.9 |
| 283 | CHEMBL562 | GRISEOFULVIN | -6.9 |
| 284 | CHEMBL599 | MELOXICAM | -6.9 |
| 285 | CHEMBL713 | ENTECAVIR | -6.9 |
| 286 | CHEMBL87992 | ESLICARBAZEPINE ACETATE | -6.9 |
| 287 | CHEMBL1055 | CHLORTHALIDONE | -6.8 |
| 288 | CHEMBL1088 | MESORIDAZINE | -6.8 |
| 289 | CHEMBL1088977 | ADEMETIONINE | -6.8 |
| 290 | CHEMBL1121 | SINCALIDE | -6.8 |
| 291 | CHEMBL1201198 | PEMIROLAST | -6.8 |
| 292 | CHEMBL1213252 | CLORAZEPIC ACID | -6.8 |
| 293 | CHEMBL1219 | RABEPRAZOLE | -6.8 |
| 294 | CHEMBL1237119 | TREPROSTINIL | -6.8 |
| 295 | CHEMBL1237132 | CLEVIDIPINE | -6.8 |
| 296 | CHEMBL129 | ZIDOVUDINE | -6.8 |
| 297 | CHEMBL1472 | PROTIRELIN | -6.8 |
| 298 | CHEMBL1569487 | LORNOXICAM | -6.8 |
| 299 | CHEMBL1789941 | RUXOLITINIB | -6.8 |
| 300 | CHEMBL225071 | RALTITREXED | -6.8 |
| 301 | CHEMBL2355051 | CLOMIPHENE | -6.8 |
| 302 | CHEMBL28218 | BROMPERIDOL | -6.8 |
| 303 | CHEMBL46469 | ANTHRALIN | -6.8 |
| 304 | CHEMBL473 | DOFETILIDE | -6.8 |
| 305 | CHEMBL522038 | XIMELAGATRAN | -6.8 |
| 306 | CHEMBL563 | FLURBIPROFEN | -6.8 |
| 307 | CHEMBL672 | FENOFIBRATE | -6.8 |
| 308 | CHEMBL781 | MAZINDOL | -6.8 |
| 309 | CHEMBL848 | LENALIDOMIDE | -6.8 |
| 310 | CHEMBL866 | MYCOPHENOLIC ACID | -6.8 |
| 311 | CHEMBL967 | TEMAZEPAM | -6.8 |
| 312 | CHEMBL1085 | ACETOPHENAZINE | -6.7 |
| 313 | CHEMBL1201772 | PRASUGREL | -6.7 |
| 314 | CHEMBL1228 | OXYPHENBUTAZONE | -6.7 |
| 315 | CHEMBL1337 | NITISINONE | -6.7 |
| 316 | CHEMBL1565476 | APAZONE | -6.7 |
| 317 | CHEMBL162036 | BENORILATE | -6.7 |
| 318 | CHEMBL187709 | TRIPARANOL | -6.7 |
| 319 | CHEMBL2107360 | PALIPERIDONE PALMITATE | -6.7 |
| 320 | CHEMBL23588 | FLUFENAMIC ACID | -6.7 |
| 321 | CHEMBL2443262 | OLICERIDINE | -6.7 |
| 322 | CHEMBL251940 | PERICIAZINE | -6.7 |
| 323 | CHEMBL267744 | TICRYNAFEN | -6.7 |
| 324 | CHEMBL461522 | DIPYRONE | -6.7 |
| 325 | CHEMBL4650319 | MOBOCERTINIB | -6.7 |
| 326 | CHEMBL617 | CEPHALOTHIN | -6.7 |
| 327 | CHEMBL62193 | SULFADIMETHOXINE | -6.7 |
| 328 | CHEMBL960 | LEFLUNOMIDE | -6.7 |
| 329 | CHEMBL1089641 | TRYPAN BLUE | -6.6 |
| 330 | CHEMBL1107 | HALOFANTRINE | -6.6 |
| 331 | CHEMBL1197051 | ISOXSUPRINE | -6.6 |
| 332 | CHEMBL1201195 | CEFMETAZOLE | -6.6 |
| 333 | CHEMBL1201320 | ESOMEPRAZOLE | -6.6 |
| 334 | CHEMBL1237 | LISINOPRIL | -6.6 |
| 335 | CHEMBL13209 | NITRAZEPAM | -6.6 |
| 336 | CHEMBL13376 | CLOMETACIN | -6.6 |
| 337 | CHEMBL16 | PHENYTOIN | -6.6 |
| 338 | CHEMBL1773 | CAPECITABINE | -6.6 |
| 339 | CHEMBL1801800 | METABROMSALAN | -6.6 |
| 340 | CHEMBL268869 | SULFAMETHOXYPYRIDAZINE | -6.6 |
| 341 | CHEMBL290960 | NIFURTIMOX | -6.6 |
| 342 | CHEMBL407 | FLUMAZENIL | -6.6 |
| 343 | CHEMBL515606 | CILAZAPRIL | -6.6 |
| 344 | CHEMBL53418 | DANTHRON | -6.6 |
| 345 | CHEMBL54349 | ALPIDEM | -6.6 |
| 346 | CHEMBL783 | NATEGLINIDE | -6.6 |
| 347 | CHEMBL1008 | BEPRIDIL | -6.5 |
| 348 | CHEMBL1051 | LATANOPROST | -6.5 |
| 349 | CHEMBL1101 | BIPERIDEN | -6.5 |
| 350 | CHEMBL1200370 | BENZOYL PEROXIDE | -6.5 |
| 351 | CHEMBL1201112 | NELARABINE | -6.5 |
| 352 | CHEMBL1208422 | ROSE BENGAL | -6.5 |
| 353 | CHEMBL154111 | SALSALATE | -6.5 |
| 354 | CHEMBL1672 | CEFPODOXIME | -6.5 |
| 355 | CHEMBL1697838 | GLYMIDINE | -6.5 |
| 356 | CHEMBL1998966 | PIFOXIME | -6.5 |
| 357 | CHEMBL2103873 | MACITENTAN | -6.5 |
| 358 | CHEMBL2105581 | VERALIPRIDE | -6.5 |
| 359 | CHEMBL2105720 | LESINURAD | -6.5 |
| 360 | CHEMBL243712 | AMISULPRIDE | -6.5 |
| 361 | CHEMBL374731 | TELBIVUDINE | -6.5 |
| 362 | CHEMBL428880 | CROMOLYN | -6.5 |
| 363 | CHEMBL509 | MECLOFENAMIC ACID | -6.5 |
| 364 | CHEMBL515914 | AZARIBINE | -6.5 |
| 365 | CHEMBL686 | MEFENAMIC ACID | -6.5 |
| 366 | CHEMBL880 | FAMCICLOVIR | -6.5 |
| 367 | CHEMBL932 | DIPYRIDAMOLE | -6.5 |
| 368 | CHEMBL93645 | ACECLOFENAC | -6.5 |
| 369 | CHEMBL1027 | TIAGABINE | -6.4 |
| 370 | CHEMBL1165268 | IODIPAMIDE | -6.4 |
| 371 | CHEMBL117287 | PRUCALOPRIDE | -6.4 |
| 372 | CHEMBL1201056 | SULFACYTINE | -6.4 |
| 373 | CHEMBL1201147 | OCTOCRYLENE | -6.4 |
| 374 | CHEMBL1201325 | HEXOCYCLIUM | -6.4 |
| 375 | CHEMBL121626 | TOLFENAMIC ACID | -6.4 |
| 376 | CHEMBL1428 | NIMODIPINE | -6.4 |
| 377 | CHEMBL1457 | HYDROCODONE | -6.4 |
| 378 | CHEMBL1503 | OMEPRAZOLE | -6.4 |
| 379 | CHEMBL1521 | ZALEPLON | -6.4 |
| 380 | CHEMBL1525826 | SULFALENE | -6.4 |
| 381 | CHEMBL1595 | DIHYDROCODEINE | -6.4 |
| 382 | CHEMBL16476 | PRONETALOL | -6.4 |
| 383 | CHEMBL2095212 | MIRABEGRON | -6.4 |
| 384 | CHEMBL2104537 | AMOPROXAN | -6.4 |
| 385 | CHEMBL2105395 | OSPEMIFENE | -6.4 |
| 386 | CHEMBL2107381 | URIDINE TRIACETATE | -6.4 |
| 387 | CHEMBL285802 | ZOTEPINE | -6.4 |
| 388 | CHEMBL30116 | PIPEMIDIC ACID | -6.4 |
| 389 | CHEMBL349803 | PIRETANIDE | -6.4 |
| 390 | CHEMBL3989949 | CENOBAMATE | -6.4 |
| 391 | CHEMBL446 | SULFAMETHAZINE | -6.4 |
| 392 | CHEMBL817 | TOLAZAMIDE | -6.4 |
| 393 | CHEMBL969 | PRAZEPAM | -6.4 |
| 394 | CHEMBL1004 | DOXYLAMINE | -6.3 |
| 395 | CHEMBL1200359 | SULFAMETER | -6.3 |
| 396 | CHEMBL1200868 | PHENYL AMINOSALICYLATE | -6.3 |
| 397 | CHEMBL1297 | FENOPROFEN | -6.3 |
| 398 | CHEMBL1331216 | HYOSCYAMINE | -6.3 |
| 399 | CHEMBL1334860 | DILOXANIDE FUROATE | -6.3 |
| 400 | CHEMBL1442422 | DIBENZEPIN | -6.3 |
| 401 | CHEMBL1648 | ISRADIPINE | -6.3 |
| 402 | CHEMBL1726 | NISOLDIPINE | -6.3 |
| 403 | CHEMBL19224 | PAPAVERINE | -6.3 |
| 404 | CHEMBL2105131 | MEFRUSIDE | -6.3 |
| 405 | CHEMBL2364639 | VALBENAZINE | -6.3 |
| 406 | CHEMBL3989859 | TOCOPHEROL ACETATE | -6.3 |
| 407 | CHEMBL438 | SULFAMERAZINE | -6.3 |
| 408 | CHEMBL488 | AMINOGLUTETHIMIDE | -6.3 |
| 409 | CHEMBL557555 | CIPROFIBRATE | -6.3 |
| 410 | CHEMBL56367 | NIMESULIDE | -6.3 |
| 411 | CHEMBL70418 | CLOBAZAM | -6.3 |
| 412 | CHEMBL93 | ZILEUTON | -6.3 |
| 413 | CHEMBL98 | VORINOSTAT | -6.3 |
| 414 | CHEMBL1086 | DIBUCAINE | -6.2 |
| 415 | CHEMBL1129 | TRIFLURIDINE | -6.2 |
| 416 | CHEMBL1164729 | FEBUXOSTAT | -6.2 |
| 417 | CHEMBL1187724 | METHYLATROPINE | -6.2 |
| 418 | CHEMBL1200910 | SULFISOXAZOLE ACETYL | -6.2 |
| 419 | CHEMBL1898387 | MEXENONE | -6.2 |
| 420 | CHEMBL1900528 | TIOTROPIUM | -6.2 |
| 421 | CHEMBL2107062 | PHENETURIDE | -6.2 |
| 422 | CHEMBL25105 | HEXAMIDINE | -6.2 |
| 423 | CHEMBL26 | SULPIRIDE | -6.2 |
| 424 | CHEMBL277062 | BROMAZEPAM | -6.2 |
| 425 | CHEMBL32800 | FENOTEROL | -6.2 |
| 426 | CHEMBL328560 | SULTHIAME | -6.2 |
| 427 | CHEMBL365795 | TIAPROFENIC ACID | -6.2 |
| 428 | CHEMBL370805 | COCAINE | -6.2 |
| 429 | CHEMBL4101807 | VOXELOTOR | -6.2 |
| 430 | CHEMBL462394 | METRIZAMIDE | -6.2 |
| 431 | CHEMBL649 | NADOLOL | -6.2 |
| 432 | CHEMBL956 | SUPROFEN | -6.2 |
| 433 | CHEMBL1079604 | METAXALONE | -6.1 |
| 434 | CHEMBL1109 | SULFAPHENAZOLE | -6.1 |
| 435 | CHEMBL1175 | DULOXETINE | -6.1 |
| 436 | CHEMBL1191 | SULFAMETHIZOLE | -6.1 |
| 437 | CHEMBL1197 | HEXYLCAINE | -6.1 |
| 438 | CHEMBL1200986 | HALOPERIDOL DECANOATE | -6.1 |
| 439 | CHEMBL1201348 | MENADIOL DIPHOSPHORIC ACID | -6.1 |
| 440 | CHEMBL1479848 | ACETOMENAPHTHONE | -6.1 |
| 441 | CHEMBL1540 | PENCICLOVIR | -6.1 |
| 442 | CHEMBL175 | DEXIBUPROFEN | -6.1 |
| 443 | CHEMBL1909285 | NITREFAZOLE | -6.1 |
| 444 | CHEMBL2181927 | FINERENONE | -6.1 |
| 445 | CHEMBL496 | HEXACHLOROPHENE | -6.1 |
| 446 | CHEMBL559 | DEXTROTHYROXINE | -6.1 |
| 447 | CHEMBL700 | SULFAPYRIDINE | -6.1 |
| 448 | CHEMBL741 | LAMOTRIGINE | -6.1 |
| 449 | CHEMBL953 | ENTACAPONE | -6.1 |
| 450 | CHEMBL106 | FLUCONAZOLE | -6 |
| 451 | CHEMBL1077896 | ROPIVACAINE | -6 |
| 452 | CHEMBL1200438 | TIOCONAZOLE | -6 |
| 453 | CHEMBL1201075 | IOXILAN | -6 |
| 454 | CHEMBL1201192 | ARMODAFINIL | -6 |
| 455 | CHEMBL1373 | MODAFINIL | -6 |
| 456 | CHEMBL1535 | HYDROXYCHLOROQUINE | -6 |
| 457 | CHEMBL15677 | FENCLOFENAC | -6 |
| 458 | CHEMBL1619 | CLADRIBINE | -6 |
| 459 | CHEMBL1631694 | FEXINIDAZOLE | -6 |
| 460 | CHEMBL1750 | CLOFARABINE | -6 |
| 461 | CHEMBL182 | GANCICLOVIR | -6 |
| 462 | CHEMBL190677 | BENZOIN | -6 |
| 463 | CHEMBL220491 | BRINZOLAMIDE | -6 |
| 464 | CHEMBL416 | METHOXSALEN | -6 |
| 465 | CHEMBL70046 | SORIVUDINE | -6 |
| 466 | CHEMBL1005 | REMIFENTANIL | -5.9 |
| 467 | CHEMBL1098 | BUPIVACAINE | -5.9 |
| 468 | CHEMBL1103 | FURAZOLIDONE | -5.9 |
| 469 | CHEMBL1483 | ALBENDAZOLE | -5.9 |
| 470 | CHEMBL1489 | AZACITIDINE | -5.9 |
| 471 | CHEMBL1542 | AZATHIOPRINE | -5.9 |
| 472 | CHEMBL1639 | ALISKIREN | -5.9 |
| 473 | CHEMBL1725 | IOPROMIDE | -5.9 |
| 474 | CHEMBL184 | ACYCLOVIR | -5.9 |
| 475 | CHEMBL188952 | PIRPROFEN | -5.9 |
| 476 | CHEMBL193 | NIFEDIPINE | -5.9 |
| 477 | CHEMBL1945778 | IOFLUPANE | -5.9 |
| 478 | CHEMBL2105345 | PROPANIDID | -5.9 |
| 479 | CHEMBL3989517 | IOFLUPANE I 123 | -5.9 |
| 480 | CHEMBL404849 | SULOCTIDIL | -5.9 |
| 481 | CHEMBL477 | ADENOSINE | -5.9 |
| 482 | CHEMBL628 | PENTOXIFYLLINE | -5.9 |
| 483 | CHEMBL782 | TOLBUTAMIDE | -5.9 |
| 484 | CHEMBL86304 | MOCLOBEMIDE | -5.9 |
| 485 | CHEMBL917 | FLOXURIDINE | -5.9 |
| 486 | CHEMBL991 | STAVUDINE | -5.9 |
| 487 | CHEMBL1193 | PHENIRAMINE | -5.8 |
| 488 | CHEMBL1201766 | FOSPROPOFOL | -5.8 |
| 489 | CHEMBL1213351 | PROPOXYPHENE | -5.8 |
| 490 | CHEMBL1480 | FELODIPINE | -5.8 |
| 491 | CHEMBL1580 | PENTOSTATIN | -5.8 |
| 492 | CHEMBL1643 | RIBAVIRIN | -5.8 |
| 493 | CHEMBL18 | ETHOXZOLAMIDE | -5.8 |
| 494 | CHEMBL1909282 | FENCLOZIC ACID | -5.8 |
| 495 | CHEMBL203321 | BRINCIDOFOVIR | -5.8 |
| 496 | CHEMBL24 | ATENOLOL | -5.8 |
| 497 | CHEMBL24944 | TRIBROMSALAN | -5.8 |
| 498 | CHEMBL316561 | PROGLUMIDE | -5.8 |
| 499 | CHEMBL456 | ETHACRYNIC ACID | -5.8 |
| 500 | CHEMBL54976 | TRYPTOPHAN | -5.8 |
| 501 | CHEMBL697 | METHSUXIMIDE | -5.8 |
| 502 | CHEMBL750 | ZONISAMIDE | -5.8 |
| 503 | CHEMBL788 | IDOXURIDINE | -5.8 |
| 504 | CHEMBL797 | PHENSUXIMIDE | -5.8 |
| 505 | CHEMBL1009 | LEVODOPA | -5.7 |
| 506 | CHEMBL1096979 | BENSERAZIDE | -5.7 |
| 507 | CHEMBL1200714 | CHLORMEZANONE | -5.7 |
| 508 | CHEMBL1237044 | TRAMADOL | -5.7 |
| 509 | CHEMBL139877 | SULFACARBAMIDE | -5.7 |
| 510 | CHEMBL290106 | BITHIONOL | -5.7 |
| 511 | CHEMBL404 | TAZOBACTAM | -5.7 |
| 512 | CHEMBL434200 | TERTATOLOL | -5.7 |
| 513 | CHEMBL45029 | MEPHOBARBITAL | -5.7 |
| 514 | CHEMBL457 | GEMFIBROZIL | -5.7 |
| 515 | CHEMBL58323 | ERLOSAMIDE | -5.7 |
| 516 | CHEMBL607710 | CHLORPHENESIN CARBAMATE | -5.7 |
| 517 | CHEMBL6995 | PRACTOLOL | -5.7 |
| 518 | CHEMBL885 | EMTRICITABINE | -5.7 |
| 519 | CHEMBL1094966 | PIRBUTEROL | -5.6 |
| 520 | CHEMBL1200604 | TROPICAMIDE | -5.6 |
| 521 | CHEMBL1460 | DIDANOSINE | -5.6 |
| 522 | CHEMBL1752 | DYPHYLLINE | -5.6 |
| 523 | CHEMBL1908307 | FLOSEQUINAN | -5.6 |
| 524 | CHEMBL463 | AMINOHIPPURIC ACID | -5.6 |
| 525 | CHEMBL861 | MEPHENYTOIN | -5.6 |
| 526 | CHEMBL1200455 | IOHEXOL | -5.5 |
| 527 | CHEMBL1200555 | IOTROLAN | -5.5 |
| 528 | CHEMBL1200932 | IOPAMIDOL | -5.5 |
| 529 | CHEMBL1201117 | METHOCARBAMOL | -5.5 |
| 530 | CHEMBL1201129 | DECITABINE | -5.5 |
| 531 | CHEMBL1201243 | IPODATE | -5.5 |
| 532 | CHEMBL1201261 | TYROPANOIC ACID | -5.5 |
| 533 | CHEMBL1329455 | MOLSIDOMINE | -5.5 |
| 534 | CHEMBL141 | LAMIVUDINE | -5.5 |
| 535 | CHEMBL1615439 | PHENDIMETRAZINE | -5.5 |
| 536 | CHEMBL2107175 | RANELIC ACID | -5.5 |
| 537 | CHEMBL498 | CHLORPROPAMIDE | -5.5 |
| 538 | CHEMBL681 | ETOMIDATE | -5.5 |
| 539 | CHEMBL1002 | LEVOSALBUTAMOL | -5.4 |
| 540 | CHEMBL1118 | DESVENLAFAXINE | -5.4 |
| 541 | CHEMBL1194 | PRILOCAINE | -5.4 |
| 542 | CHEMBL1198 | PRAMOXINE | -5.4 |
| 543 | CHEMBL1474889 | CLOBUTINOL | -5.4 |
| 544 | CHEMBL1681 | IODOHIPPURATE | -5.4 |
| 545 | CHEMBL201960 | PYROVALERONE | -5.4 |
| 546 | CHEMBL314854 | FINGOLIMOD | -5.4 |
| 547 | CHEMBL46917 | CARBARIL | -5.4 |
| 548 | CHEMBL500 | PINDOLOL | -5.4 |
| 549 | CHEMBL810 | TEMOZOLOMIDE | -5.4 |
| 550 | CHEMBL859 | OXYPURINOL | -5.4 |
| 551 | CHEMBL902 | FAMOTIDINE | -5.4 |
| 552 | CHEMBL925 | TYROSINE | -5.4 |
| 553 | CHEMBL1159717 | TULOBUTEROL | -5.3 |
| 554 | CHEMBL1195 | PROPOXYCAINE | -5.3 |
| 555 | CHEMBL1410 | NONOXYNOL 9 | -5.3 |
| 556 | CHEMBL1697856 | BUCETIN | -5.3 |
| 557 | CHEMBL1790041 | RANITIDINE | -5.3 |
| 558 | CHEMBL1179047 | CHLOROPROCAINE | -5.2 |
| 559 | CHEMBL1184360 | DIAMTHAZOLE | -5.2 |
| 560 | CHEMBL1201131 | PIPERONYL BUTOXIDE | -5.2 |
| 561 | CHEMBL1615487 | IODOXAMIC ACID | -5.2 |
| 562 | CHEMBL1987462 | BAMETHAN | -5.2 |
| 563 | CHEMBL2104613 | OCTOXYNOL 9 | -5.2 |
| 564 | CHEMBL2105002 | ISOAMINILE | -5.2 |
| 565 | CHEMBL284906 | NICORANDIL | -5.2 |
| 566 | CHEMBL455 | SULFACETAMIDE | -5.2 |
| 567 | CHEMBL1194666 | DIETHYLPROPION | -5.1 |
| 568 | CHEMBL1196 | PROPARACAINE | -5.1 |
| 569 | CHEMBL1200770 | IOCETAMIC ACID | -5.1 |
| 570 | CHEMBL1232131 | DOBESILIC ACID | -5.1 |
| 571 | CHEMBL853 | ZALCITABINE | -5.1 |
| 572 | CHEMBL1093 | ARTICAINE | -5 |
| 573 | CHEMBL1200 | BENOXINATE | -5 |
| 574 | CHEMBL1469 | PHENYLBUTANOIC ACID | -5 |
| 575 | CHEMBL190 | THEOPHYLLINE | -5 |
| 576 | CHEMBL1909286 | PHENOXYPROPAZINE | -5 |
| 577 | CHEMBL229128 | MEPHENESIN | -5 |
| 578 | CHEMBL37390 | PROXYPHYLLINE | -5 |
| 579 | CHEMBL1200614 | IOVERSOL | -4.9 |
| 580 | CHEMBL435966 | NIMORAZOLE | -4.9 |
| 581 | CHEMBL867 | IOPANOIC ACID | -4.9 |
| 582 | CHEMBL980 | GUAIFENESIN | -4.9 |
| 583 | CHEMBL113 | CAFFEINE | -4.8 |
| 584 | CHEMBL1201786 | LAPYRIUM | -4.8 |
| 585 | CHEMBL1220 | TINIDAZOLE | -4.8 |
| 586 | CHEMBL1449676 | ORNIDAZOLE | -4.8 |
| 587 | CHEMBL704 | MESALAMINE | -4.8 |
| 588 | CHEMBL108545 | METHYL SALICYLATE | -4.7 |
| 589 | CHEMBL1115 | PYRIDOSTIGMINE | -4.7 |
| 590 | CHEMBL1374379 | BENZONATATE | -4.6 |
| 591 | CHEMBL498847 | SECNIDAZOLE | -4.5 |
| 592 | CHEMBL1229846 | PHENOXYETHANOL | -4.3 |
